# Changes in mean and variance of ophthalmic disease incidences during COVID-19 pandemic in Korea

**DOI:** 10.1038/s41598-022-24975-z

**Published:** 2022-11-27

**Authors:** Hyo Geun Choi, So Young Kim, Sung Uk Baek

**Affiliations:** 1 Hallym Data Science Laboratory, Hallym University College of Medicine, Anyang, Korea; 2 Department of Otorhinolaryngology-Head & Neck Surgery, Hallym University College of Medicine, Hallym University Sacred Heart Hospital, Anyang, Korea; 3 Department of Otorhinolaryngology-Head & Neck Surgery, CHA Bundang Medical Center, CHA University, Seongnam, Korea; 4 Department of Ophthalmology, Hallym University College of Medicine, Hallym University Sacred Heart Hospital, 22, Gwanpyeong-Ro 170Beon-Gil, Dongan-Gu, Anyang-Si, Gyeonggi-Do 14068 South Korea

**Keywords:** Diseases, Eye diseases, Public health

## Abstract

This study undertook to determine the changes in the numbers of outpatient visits for various ophthalmic diseases during the COVID-19 pandemic compared with before. The monthly outpatient visits for 14 common ophthalmic diseases were enumerated based on the ICD-10 codes in Korean National Health Insurance Service data. The differences in the mean outpatient visits and disease variance ‘before’ and ‘during COVID-19’ were calculated. Subsequently, subgroup analyses according to age and sex were performed. The number of outpatient visits for conjunctivitis, scleritis & episcleritis, keratitis, cataract, diabetic retinopathy, and traumatic ophthalmic disease were lower during than before COVID-19 (all P < 0.001). The lower numbers of outpatient visits for ophthalmic disorders during COVID-19 were consistent across the age and sex subgroups. All ophthalmic diseases other than endophthalmitis showed no change of variation ‘during’ relative to ‘before’ COVID-19. In conclusion, during the COVID-19 pandemic, the ophthalmic outpatient visits decreased for infectious and inflammatory diseases, screening diseases, and traumatic diseases. However, COVID-19 is not considered to have had a significant effect on variation.

## Introduction

The Coronavirus disease 2019 (COVID-19) pandemic was an unprecedented global event that changed health care and social landscapes. It necessitated remarshalling of medical resources in order to triage emergent diseases^1^. Due to the limitations imposed on clinic accessibility, services and procedures were preferentially provided to those with emergent or acute phases of diseases. Thus, concerns about healthcare inequality were and continue to be raised^2,3^.

In addition to medical resources, the epidemiologic features of diseases also were impacted during COVID-19. Changes in health-seeking behavior and improved hygiene had, and continue to have, an effect on disease incidence^4^. A healthy diet and good lifestyle habits have been suggested to decrease the incidence of metabolic as well as infectious diseases. Besides, the long-lasting COVID-19 pandemic impaired social activities and affected economic status due to the quarantine strategies of social distancing and lockdown of industries^5^. Thus, it can be supposed that clinic accessibility may have been reduced and that people may have been reluctant to visit medical facilities in cases where they judged their situation to be ‘not serious’.

Given that ophthalmic diseases include many infectious and inflammatory, neurogenerative, and traumatic disorders, their incidences were proved, by two studies, to be especially sensitive to the COVID-19 pandemic^6,7^. One of those study populations, however, was limited to children, and was relatively small (4,538) as well^6^. Also, there is still no research on the variance or seasonality of ophthalmic diseases during COVID-19.

Thus, the impacts of COVID-19 on a wide range of ophthalmic diseases needed to be evaluated in a large population cohort. Furthermore, we thought the actual numbers of outpatient visits might differ from survey results. Few previous studies have extensively investigated the impact of COVID-19 on the epidemiology of ophthalmic diseases in large, nationwide populations.

We hypothesized that the incidence and variance of a wide range of ophthalmic diseases, in addition to conjunctivitis and traumatic diseases, may have been altered during the COVID-19 era. To test these hypotheses, in the present study, we compared the number and variation of outpatient visits for numerous ophthalmic diseases between ‘before-’ versus ‘during COVID-19.’ Further, to estimate the seasonality of diseases, outpatient visits were enumerated monthly and compared.

## Results

Outpatients’ visits for conjunctivitis, scleritis & episcleritis, keratitis, cataract, diabetic retinopathy, and traumatic ophthalmic disease were lower during COVID-19 than before (all P < 0.001, Table 1). Meanwhile, retinal detachment, retinal vascular occlusion, glaucoma, and endophthalmitis incidences were higher during COVID-19 than before (all P < 0.001, Table 1). All of the ophthalmic diseases other than endophthalmitis showed no change of variation in disease incidence during COVID-19 compared with before (all P > 0.05, Table 1).

**Table 1:** Means, standard deviations of disease incidence before and during COVID-19 and their differences.

Diseases	Before COVID-19		During COVID-19		P-values of difference	
	Mean	SD	Mean	SD	Mean	Variance
**Infectious and inflammatory ophthalmic diseases**						
Inflammatory eyelid disease	205,149.3	20,220.5	207,333.7	17,465.3	0.685	0.620
Conjunctivitis	549,683.4	97,460.5	469,450.7	80,529.4	0.020*	0.846
Scleritis & episcleritis	7609.3	430.8	7183.3	571.4	0.010*	0.211
Keratitis	230,857.1	14,676.2	195,330.5	22,408.7	< 0.001*	0.414
Anterior uveitis	14,089.8	929.5	14,192.3	952.4	0.725	0.805
Intermediate and posterior uveitis	1813.7	102.1	1759.6	91.3	0.123	0.319
Endophthalmitis	1058.8	75.7	1275.9	243.7	0.014*	0.005†
Optic neuropathy	1034.4	75.7	1052.8	91.0	0.372	0.173
**Non-infectious and non-inflammatory ophthalmic diseases**						
Cataract	252,491.9	18,383.4	232,035.7	23,971.7	0.007*	0.211
Retinal detachment	14,386.6	1075.8	15,545.7	1112.1	0.001*	0.211
Retinal vascular occlusion	16,681.8	1232.6	17,979.3	1118.6	0.003*	0.697
Diabetic retinopathy	90,698.3	5215.5	86,414.9	5138.0	0.011*	0.736
Glaucoma	197,147.4	11,726.5	207,709.5	11,971.5	< 0.001*	0.295
Traumatic ophthalmic disease	38,988.7	3222.3	33,258.5	2269.1	< 0.001*	0.697

*Mann Whitney *U* test, significance at < 0.05.

†Levene’s test for non-parametric data, significance at < 0.05.

**Figure 1:**
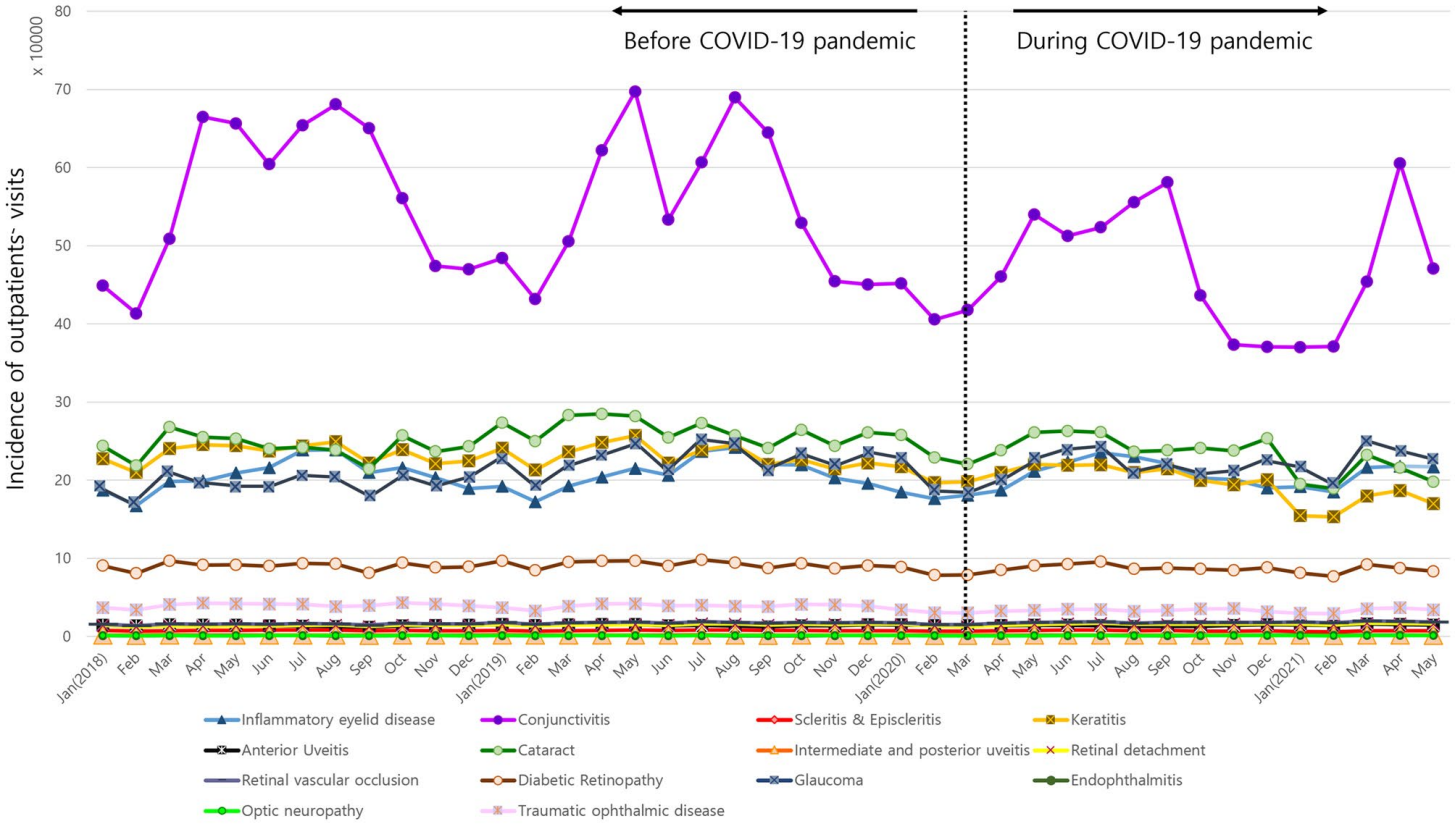
Distribution of various ophthalmic disease incidences (i.e., outpatient visits) before and during COVID-19 pandemic.

**Figure 2:**
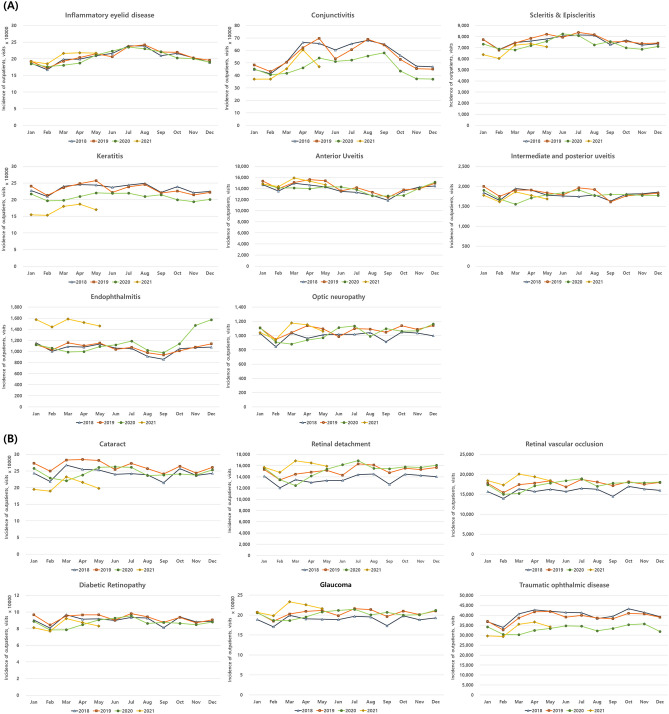
Monthly ophthalmic disease incidences (i.e., outpatient visits) during 2018, 2019, 2020, and 2021. The incidences of (**A**) *infectious and inflammatory ophthalmic diseases* (i.e., inflammatory eyelid disease, conjunctivitis, scleritis & episcleritis, keratitis, anterior uveitis, intermediate and posterior uveitis, endophthalmitis, and optic neuropathy) and (**B**) *non-infectious and non-inflammatory ophthalmic diseases* (i.e., cataract, retinal detachment, retinal vascular occlusion, diabetic retinopathy, glaucoma, and traumatic ophthalmic disease) are plotted.

Most of the infectious and inflammatory diseases did not show seasonal changes of incidence, but some, namely conjunctivitis, keratitis, and inflammatory eyelid disease, showed seasonality before COVID-19 (Fig. 1). In these diseases, the seasonality was generally maintained even after COVID-19. The incidence of conjunctivitis was higher during spring and summer and lower during autumn and winter before COVID-19 (Fig. 2). The incidence of inflammatory eyelid disease was higher in summer and lower in winter. Similarly, keratitis showed a low incidence in winter. Among non-infectious and non-inflammatory diseases, seasonal variation was not evident even before COVID-19, and this pattern remained constant during COVID-19 (Fig. 1).

According to sex, both the male and female groups showed no during-versus-before-COVID-19 difference of incidence for conjunctivitis, keratitis, cataract, diabetic retinopathy, traumatic ophthalmic disease, retinal detachment, retinal vascular occlusion or glaucoma (all P < 0.05, Table 2). Only the female group showed decreased incidence for scleritis & episcleritis during relative to before COVID-19 (all P < 0.05, Table 2). Additionally, in the male group, endophthalmitis showed higher incidence during than before COVID-19. Variance of disease incidence did not differ before versus during COVID-19 for either the male or the female group (all P > 0.05), except for endophthalmitis. As for age, cataract seasonality showed different patterns before versus during COVID-19in the 20–59 years group: specifically, higher monthly variability during than before (Table 3). In the ≥ 60 years group, the variance increased for endophthalmitis during COVID-19.

**Table 2:** Means, standard deviations of disease incidence before and during COVID-19 and their differences by sex.

Diseases	Before COVID-19		During COVID-19		P-values of Difference	
	Mean	SD	Mean	SD	Mean	Variance
**Men**						
Infectious and inflammatory ophthalmic diseases						
Inflammatory eyelid disease	84,924.0	8949.5	86,351.7	7464.7	0.626	0.764
Conjunctivitis	225,402.4	46,042.2	192,030.0	38,668.5	0.037*	0.972
Scleritis & episcleritis	3473.0	269.8	3353.5	346.6	0.291	0.301
Keratitis	81,623.1	6450.8	70,823.5	8916.5	< 0.001*	0.658
Anterior uveitis	7594.9	466.4	7548.5	437.5	0.892	0.225
Intermediate and posterior uveitis	871.2	44.4	828.4	37.7	0.003*	0.972
Endophthalmitis	510.8	29.6	617.1	95.0	< 0.001*	0.034†
Optic neuropathy	464.1	33.3	456.5	33.2	0.401	0.943
Non-infectious and non-inflammatory ophthalmic diseases						
Cataract	101,838.8	6494.5	93,072.4	10,134.1	0.008*	0.127
Retinal detachment	7375.9	516.6	7791.8	488.5	0.009*	0.413
Retinal vascular occlusion	7184.6	512.0	7845.7	470.4	< 0.001*	0.355
Diabetic retinopathy	43,391.1	2296.0	42,017.6	2298.8	0.037*	0.697
Glaucoma	94,242.2	5489.1	99,369.5	5122.7	0.049*	0.748
Traumatic ophthalmic disease	25,301.4	2324.4	21,137.1	1568.0	< 0.001*	0.859
**Women**						
Infectious and inflammatory ophthalmic diseases						
Inflammatory eyelid disease	120,225.4	11,312.1	120,982.1	10,155.8	0.829	0.658
Conjunctivitis	324,281.0	51,686.5	277,420.7	42,434.8	0.012*	0.888
Scleritis & episcleritis	4136.3	194.0	3829.8	238.5	< 0.001*	0.211
Keratitis	149,234.0	9086.5	124,507.0	13,732.5	< 0.001*	0.114
Anterior uveitis	6494.9	469.4	6643.8	530.4	0.457	0.964
Intermediate and posterior uveitis	942.5	66.0	931.2	61.5	0.745	0.346
Endophthalmitis	548.1	57.6	658.8	149.7	0.074	0.027†
Optic neuropathy	570.3	48.3	596.3	60.5	0.194	0.156
Non-infectious and non-inflammatory ophthalmic diseases						
Cataract	150,653.2	12,165.0	138,963.3	14,105.3	0.023*	0.736
Retinal detachment	7010.7	571.2	7753.9	634.5	< 0.001*	0.101
Retinal vascular occlusion	9497.2	732.3	10,133.6	665.2	0.010*	0.888
Diabetic retinopathy	47,307.2	2975.2	44,397.3	2886.3	0.005*	0.986
Glaucoma	102,905.3	6302.5	108,345.0	6941.9	0.001*	0.628
Traumatic ophthalmic disease	13,687.3	972.3	12,121.3	750.1	< 0.001*	0.818

* Mann Whitney *U* test, significance at < 0.05.

†Levene’s test for non-parametric data, significance at < 0.05.

**Table 3:** Means, standard deviations of disease incidence before and during COVID-19 and their differences by age.

Diseases	Before COVID-19		During COVID-19		P-values of difference	
	Mean	SD	Mean	SD	Mean	Variance
**Age 0–19 years**						
Infectious and inflammatory ophthalmic diseases						
Inflammatory eyelid disease	61,843.8	8492.2	61,490.4	7164.9	0.892	0.506
Conjunctivitis	149,469.5	44,804.2	105,531.9	31,028.2	0.003*	0.810
Scleritis & episcleritis	361.7	113.9	295.5	111.0	0.062	0.551
Keratitis	23,744.7	4356.4	17,123.7	2841.5	< 0.001*	0.183
Anterior uveitis	427.4	41.9	409.3	54.0	0.228	0.794
Intermediate and posterior uveitis	38.2	6.6	35.7	6.8	0.139	0.997
Endophthalmitis	16.5	6.6	24.5	4.8	0.001*	0.194
Optic neuropathy	99.9	20.0	96.3	12.9	0.935	0.372
Non-infectious and non-inflammatory ophthalmic diseases						
Cataract	246.2	56.4	130.7	99.2	< 0.001*	0.804
Retinal detachment	454.7	132.6	416.9	90.0	0.636	0.083
Retinal vascular occlusion	15.0	4.8	14.7	4.2	0.957	0.824
Diabetic retinopathy	343.8	58.6	289.3	50.0	0.004*	0.595
Glaucoma	2183.7	344.3	1938.7	390.7	0.102	0.001
Traumatic ophthalmic disease	11,681.8	2093.1	8223.7	1299.4	< 0.001*	0.162
**Age 20–59 years**						
Infectious and inflammatory ophthalmic diseases						
Inflammatory eyelid disease	120,258.9	10,560.2	119,998.5	9315.3	0.914	0.507
Conjunctivitis	238,466.3	38,373.3	205,182.8	34,599.5	0.017*	0.593
Scleritis & episcleritis	5110.2	298.4	4761.4	388.9	0.005*	0.878
Keratitis	118,487.9	6855.6	97,716.6	11,022.8	< 0.001*	0.065
Anterior uveitis	8612.7	547.2	8638.4	538.4	0.766	0.554
Intermediate and posterior uveitis	1047.3	55.1	991.6	59.0	0.009*	0.555
Endophthalmitis	315.1	17.9	395.7	69.2	< 0.001*	0.678
Optic neuropathy	570.5	39.1	566.8	57.4	0.766	0.075
Non-infectious and non-inflammatory ophthalmic diseases						
Cataract	43,479.7	3015.4	40,497.7	7934.4	0.665	0.042†
Retinal detachment	8543.2	591.0	9182.2	612.7	0.001*	0.847
Retinal vascular occlusion	4414.7	282.9	4493.3	226.5	0.379	0.695
Diabetic retinopathy	27,099.0	1451.1	25,043.3	1464.0	< 0.001*	0.192
Glaucoma	71,391.0	3646.4	71,496.3	4205.9	0.002*	0.787
Traumatic ophthalmic disease	18,521.8	981.0	16,411.7	765.3	< 0.001*	0.059
**Age 60 + years**						
Infectious and inflammatory ophthalmic diseases						
Inflammatory eyelid disease	120,258.9	10,560.2	119,998.5	9315.3	0.001*	0.452
Conjunctivitis	238,466.3	38,373.3	205,182.8	34,599.5	0.685	0.498
Scleritis & episcleritis	5110.2	298.4	4761.4	388.9	0.725	0.314
Keratitis	118,487.9	6855.6	97,716.6	11,022.8	0.021*	0.545
Anterior uveitis	8612.7	547.2	8638.4	538.4	0.552	0.472
Intermediate and posterior uveitis	1047.3	55.1	991.6	59.0	0.715	0.943
Endophthalmitis	315.1	17.9	395.7	69.2	0.078	0.009†
Optic neuropathy	570.5	39.1	566.8	57.4	0.037*	0.933
Non-infectious and non-inflammatory ophthalmic diseases						
Cataract	43,479.7	3015.4	40,497.7	7934.4	0.005*	0.949
Retinal detachment	8543.2	591.0	9182.2	612.7	0.003*	0.975
Retinal vascular occlusion	4414.7	282.9	4493.3	226.5	0.001*	0.616
Diabetic retinopathy	27,099.0	1451.1	25,043.3	1464.0	0.07	0.876
Glaucoma	123,661.6	8539.8	134,359.0	7810.0	0.013*	0.257
Traumatic ophthalmic disease	18,521.8	981.0	16,411.7	765.3	0.386	0.956

*Mann Whitney *U* test, significance at < 0.05.

†Levene’s test for non-parametric data, significance at < 0.05.

## Discussion

The current study extends the previous knowledge on the impact of the COVID-19 pandemic on ophthalmic disorders by addressing the changes of incidence of a wide range of ophthalmic disorders in a nationwide population based on real-world data. During COVID-19, the number of outpatient visits decreased for infectious and inflammatory diseases, ocular screening exams including cataract and diabetic retinopathy, and traumatic disease. However, the seasonality of most of the ophthalmic diseases was not altered during COVID-19.

In this study, the decreased incidence of infectious and inflammatory diseases including conjunctivitis, scleritis & episcleritis, and keratitis was found to be related to the strengthening of hygiene management and social distancing. In personal aspects, self-protective practices during COVID-19 included wearing of facial masks, maintenance of hand hygiene and voluntary isolation at home^8,9^. In social aspects, social distancing actions encompassed school closures, working from home, telecommuting, and avoidance of mass public gatherings^10,11^. It is judged that these personal and social measures during COVID-19 lowered the incidence of inflammatory and infectious ophthalmic diseases.

The decrease in cataract and diabetic retinopathy incidences is thought to have been influenced by reduced screening testing^7,12^. Declines in both overall visits for these ophthalmic diseases may also be explained by the reluctance of patients to seek eye care for fear of exposure to COVID-19^13^. Lesser availability of medical care during COVID-19 could have negatively impacted on outpatient visits as well. Meanwhile, the decreased incidence of traumatic ophthalmic disease was judged to have been the result of fewer outside activities (e.g., sports and recreation) and lesser interpersonal contact due to social distancing^14,15^.

In line with the current results, many recent studies have demonstrated decreased incidence and prevalence of ophthalmic disorders. For example, a retrospective analysis of changes in ophthalmic patient volumes in the 6 weeks before and after COVID-19, based on the ICD-10 code, was conducted in the United States^7^. In that study, the total number of visits decreased four-fold after implementation of clinic changes associated with COVID-19. Babu et al. evaluated the effect of COVID-19 and national lockdown on tertiary-care ophthalmology in India^16^. They reported that the number of outpatient visits, retinal laser procedures, intravitreal injections and cataract surgeries during the lockdown decreased by 96.5, 96.5, 98.7 and 99.7%, respectively, compared with the corresponding time the previous year.

Interestingly, among the findings of the present study, the numbers of outpatient visits for retinal detachment, retinal vascular occlusion, glaucoma, and endophthalmitis were higher during than before COVID-19. Glaucoma outpatients’ visits increased in frequency significantly during COVID-19, reflecting the continued need for in-person intraocular pressure monitoring^17^. Environmental changes during COVID-19 and the direct effects of COVID-19 on physical health may have contributed to those increased numbers. There is also a possibility that heightened concerns about personal health due to COVID-19 made people more aware of the importance of proactive screening testing. Additionally, it is plausible that the extra time freed up for individual activities as a result of telecommuting and social distancing gave people more opportunities to seek out medical services^10^.

Among ophthalmic diseases, conjunctivitis, keratitis, and inflammatory eyelid disease show seasonal fluctuations, which is thought to be chiefly a phenomenon of summertime infectious disease outbreaks^18–20^. This seasonality showed a similar pattern in the present study during COVID-19, with no significant differences. Infectious diseases also showed similar incidence variances during COVID-19, contrary to the decreased number of outpatient visits. The high incidence of infectious ophthalmic diseases during the summer season is attributable to a number of viral and host factors, among which are increased viral stability and transmissibility coupled with weakened host immune responses^21^.

The incidence and variance of most ophthalmic diseases during COVID-19 was consistent across our age subgroups. Characteristically, in the case of the 20–59 years subgroup and cataracts, there was slightly more monthly variability during COVID-19. In accounting for this, we should consider that along with the natural age-related differences in disease incidence, there may be differences in disease awareness and treatment adherence^22^. In addition, these findings might be related to the social distancing policy that came into effect during COVID-19, because the 20–59 years subgroup is the most socially active. Especially, as the number of COVID-19 infection cases increased, the social distancing policy became more restrictive, and the resultingly fewer hospital visits might have affected a decrease in the number of cataract diagnoses.

In the present analysis according to sex, most of the ophthalmic diseases showed no significant difference in outpatient visits or variance before and during COVID-19. In the case of scleritis & episcleritis, only women showed a decreased incidence during COVID-19. Development of these diseases is known to be associated with stress and alcohol consumption^23,24^; and during COVID-19, it is likely that an increase in telecommuting, a decrease in workplace gatherings and a decrease in alcohol consumption contributed to the decreased incidence of scleritis, especially among women.

Notably, the incidence of retinal detachment and endophthalmitis significantly increased despite COVID-19. It is difficult to explain the exact reason for this, though immunological changes consequent upon COVID-19 vaccination may have played a role^25^. In addition, the nationwide campaigns and information on the COVID-19 pandemic could evoke COVID-19-related fear^26^, and furthermore, patients’ perceptions of other diseases also may change. In the case of severe diseases with the potential for blindness, such as retinal detachment, awareness has increased, and it is presumed that outpatient visits for related symptoms also have increased. In the case of endophthalmitis, the increased incidence of infectious endophthalmitis after cataract surgery in Korea could be considered in the light of the temporal relationship. Post-event analysis confirmed that the ophthalmic viscosurgical devices used during surgery were major sources of infection^27^. However, it was judged that additional analysis and research are needed to determine whether COVID-19 infection indirectly affects the pathophysiological development of retinal detachment and endophthalmitis.

Moreover, living in isolation and quarantine during COVID-19 led to increases in the symptoms of many psychiatric disorders including depression, anxiety, and stress disorder^28,29^. These psychological factors can also influence a patient's disease ecology, which can affect medical visits as well. For example, patients with mild eye disease might tolerate symptoms and avoid hospital visits. In addition to the detrimental effect on the general public’s mental health, it has been shown that frontline healthcare workers experienced significant distress during COVID-19^30^. This medical environment might change the doctor-patient relationship and management patterns for patients, which could in turn affect the prevalence and incidence of diseases.

## Limitations

There are several limitations to our study, including its retrospective design and its reliance on the ICD-10 codes for categorization of diagnoses and subclassification of ophthalmic diseases. As this study was based on health claim data that classifies diseases according to diagnostic codes, undiagnosed or subclinical diseases could have been missed. Furthermore, neither comorbid conditions nor socioeconomic status could be considered in this study. Because the COVID-19 pandemic has had impacts on various comorbidities including socioeconomic status, changes of these factors could mediate differences in outpatient visits for ophthalmic diseases^1^. Lastly, this study analyzed what amounted to, at least statistically, a single ethnic population, that of Korea. And in Korea, the COVID-19 infection rate (< 1% of the total population) was significantly lower than in the US or European countries during the study period (until May 2021)^31^. Thus, given that medical-services accessibility was not curtailed in Korea to the same extents as in other countries during COVID-19, ophthalmic disease incidence trends could have differed as well.

## Conclusions

Our findings showed that the COVID-19 pandemic has suppressed the incidence of some ophthalmic diseases. Therefore, clinicians should consider placing greater emphasis on follow-up visits and medication adherence as well as providing information to patients about alternative ways to ensure continued access to medications. Another COVID-19 mutation or an entirely new type of infectious disease might appear in the future, perhaps instigating another global crisis. The ophthalmic disease trends identified in this paper as having occurred during the COVID-19 pandemic could prove valuable to health policy making and patient management. Academic ophthalmology departments, hospitals and clinics alike should be prepared to be confronted with pandemic-related patient-volume decreases and treatment-protocol changes. Knowing the changes specific to each clinical subspecialty would be vital to correctly redistributing available resources.

## Methods

### Ethics

The present study protocol was reviewed and approved by the Institutional Review Board (IRB) of Hallym University Sacred Heart Hospital. The requirement to obtain written informed consent was waived by the IRB of Hallym University Sacred Heart Hospital, as our study was retrospective in nature and presented no more than minimal risk of harm to subjects. All methods were performed in accordance with the relevant guidelines and regulations.

### Participants and measurements

The study population was derived from the Korean National Health Insurance Service (NHIS). This database had already been used multiple times for a wide variety of epidemiologic studies, and its validity has been described in detail elsewhere^32,33^. The database covers all health claims, including those submitted through the public health insurance scheme that covers 97% of the Korean population (~ 50 million people). Thus, in the present study, we could gather data on all visits to institutions ranging from primary clinics to tertiary hospitals. Specifically, we looked at outpatient visits that had been recorded between Jan 2018 and May 2021. As the first COVID-19 patients were identified on 20 January 2020 in Korea and disease prevention and control started in March 2020, we defined the ‘before COVID-19’ period as continuing until February 2020 and the ‘during COVID-19’ period as starting from March 2020.

We scrutinized the monthly outpatient visits for 14 ophthalmic diseases commonly treated in primary clinics^34^. The patients were diagnosed with those diseases based on the ICD-10 codes. The *infectious and inflammatory ophthalmic diseases* considered were inflammatory eyelid disease (H000, H001, H018. H019), conjunctivitis (H10), scleritis & episcleritis (H150, H151, H190), keratitis (H16), anterior uveitis (H20), intermediate and posterior uveitis (H30), endophthalmitis (H440, H441), and optic neuropathy (H46, H481). Next, the *non-infectious and non-inflammatory ophthalmic diseases* considered were cataract (H25, H26), retinal detachment (H33), retinal vascular occlusion (H34), diabetic retinopathy (H350, H352, H360), glaucoma (H40), and traumatic ophthalmic disease (H210. S001, S002, S051, S011. S050, S052 ~ 059, S023, S027).

Disease incidence was calculated without duplication, as we had access to the medical records of the entire hospital or clinic, and patients were each identified by a unique resident registration number^35,36^. To select participants who received a diagnosis of specific ophthalmic disease for the first time during the follow-up period, those diagnosed in 2016 or 2017 were excluded in consideration of the washout period. For the incidence estimates, the date of the earliest claim with a registration code was defined as the index date and was considered as the incident time, the patient being considered an incident case in that year.

### Statistics

The differences in the mean outpatient visits for diseases before and during COVID-19 were compared using the Mann–Whitney *U* test for non-parametric values. The difference in the variance of diseases before and during COVID-19 was compared using Levene’s test for non-parametric values^37^. For subgroup analyses, we divided the participants by age (0–19 years, 20–59 years, 60 + years) and sex. Two-tailed analyses were conducted, and P values < 0.05 were considered to indicate significance. The results were statistically analyzed using SPSS version 22.0 (IBM, Armonk, NY, USA).

## Data Availability

Data supporting the findings of the current study are available from the corresponding author on reasonable request.
